# **Artificial Neural Networks Analysis Used to Evaluate the Molecular Interactions between Selected Drugs and Human Cyclooxygenase2 Receptor**

**Published:** 2013-11

**Authors:** Ali Tayarani, Ali Baratian, Mohammad-Bagher Naghibi Sistani, Mohammad Reza Saberi, Zeinab Tehranizadeh

**Affiliations:** 1 Department of Electrical Engineering, Ferdosi University of Mashad, Mashad, Iran; 2 School of Pharmacy, Mashhad University of Medical Sciences, Mashad, Iran

**Keywords:** Artificial Neural Networks, Binding Energy, Cyclooxygenase 2, COX2, Docking

## Abstract

***Objective(s): ***A fast and reliable evaluation of the binding energy from a single conformation of a molecular complex is an important practical task. Artificial neural networks (ANNs) are strong tools for predicting nonlinear functions which are used in this paper to predict binding energy. We proposed a structure that obtains binding energy using physicochemical molecular descriptions of the selected drugs.

***Material and Methods:*** The set of 33 drugs with their binding energy to cyclooxygenase enzyme (COX2) in hand, from different structure groups, were considered. 27 physicochemical property descriptors were calculated by standard molecular modeling. Binding energy was calculated for each compound through docking and also ANN. A multi-layer perceptron neural network was used.

***Results:*** The proposed ANN model based on selected molecular descriptors showed a high degree of correlation between binding energy observed and calculated. The final model possessed a 27-4-1 architecture and correlation coefficients for learning, validating and testing sets equaled 0.973, 0.956 and 0.950, respectively.

*Conclusion:* Results show that docking results and ANN data have a high correlation. It was shown that ANN is a strong tool for prediction of the binding energy and thus inhibition constants for different drugs in very short periods of time.

## Introduction

Docking is a method which predicts the preferred orientation of one molecule to a second when bound to each other to form a stable complex (1). Docking is frequently used to predict the binding orientation of small molecule drug candidates to their protein targets in order to in turn predict the affinity and activity of the small molecule. Hence docking plays an important role in the rational design of drugs (2). Given the biological and pharmaceutical significance of molecular docking, considerable efforts have been directed towards improving the methods used to predict docking.

Two approaches are generally used for docking calculations. One approach uses a matching technique that describes the protein and the ligand as complementary surfaces (3). The second approach simulates the actual docking process in which the ligand-protein pairwise interaction energies are calculated (4).

In geometric matching the protein and ligand are described as sets of features that enable them to be docked. In one method receptor’s surface is described in terms of solvent accessible surface area and the ligand’s molecular surface is described in terms of matching surface description. Another method is to describe hydrophobic features of the protein using turns in main chain atoms. Yet another approach is to use a Fourier shape descriptor technique (5, 6).

The simulation of docking is a much more complicated process. In this method ligand and receptor are positioned in a distance and the ligand is let to find its way into the active site with certain number of moves. The moves incorporate rigid body transformations such as translations and rotations. 

After each move total energy of the system is calculated.

The Artificial neural network (ANN) analysis is a method of data analysis, which imitates the human brain’s way of working. The power of ANN_s_ has been shown over the years by their successful use in many types of problems with different degrees of complexity and in different fields of application. Neural networks represent the way in which arrays of neurons probably function in biological learning and memory (7). These networks are known as the universal approximations and computational models with particular characteristics such as the ability to learn or adapt, to organize or to generalize data. The learning of ANNs takes place by training with examples, “in a process that uses a training algorithm to iteratively adjust the connection weights between neurons to produce the desired input–output relationships” (8). It has been widely used in optimization, calibration, modeling and pattern recognition. ANNs are very useful in medical and pharmaceutical sciences, for example in diagnosis of diseases (). Also ANNs have shown a good potential in calculation of physic-chemical and biological properties of drugs with more attention to pharmaceutical and chemical areas (12). In recent years many studies have been done in this field. Agatonovic-Kustrin and Beresford (13) reviewed the pharmaceutical applications of ANN method. ANN has been used to calculate aqueous solubility of drugs employing a number of molecular descriptors (14), and in other situations (15-18).

It is proposed that by using artificial neural networks a set of descriptors can be incorporated to predict binding energy of final docking complex to facilitate and speed up screening processes.

The aim of this study was to design and test the appropriate ANN, which could allow predicting binding energy on basis of structural descriptors describing the structure of the selected basic drugs. 

## Materials and Methods

*Structural parameters from molecular modeling*

Descriptors of the structure of drugs were calculated by standard molecular modeling. Hyperchem® Ver. 8.5 for Windows® operating system was used. Geometry optimization was performed using molecular mechanics MM+ force field method and was followed by quantum chemical calculations according to semi-empirical AM1 method. Moreover, the set of structural descriptors was supplemented with Dragon Ver 4.5 software. The list of descriptors is presented in Table 3. 

*Docking*

Autodock Ver 4.2 on Ubuntu Linux platform was used for docking. MGL tools Ver 1.5.4 was used for preparation and conversion of structures in Linux. COX2 (PDB ID: 6COX) was used as macromolecule and was set to rigid. The grid box was created with default 40x40x40 dots, each dot being 0.375Å, and was centered in the active site of the protein guided by presence of Celecoxib in original file. Number of general algorithm (GA) runs was set to twenty and the best result of each set with lowest binding energy was chosen. Structures were finally observed and examined using Swiss PDB Viewer Ver 4.0.4 and ViewerLite 4.2 in Windows 7.

*Artificial neural network (ANN) analysis*

An ANN involves the nodes that are known as neurons. The neurons are structured into a sequence of layers and connected to each other by using variable connection weights (12). Each layer can have a number of different neurons with various transfer functions (19). The first layer is the input layer with 27 nodes. The last layer is the output layer consisting of one node and a hidden layer containing 4 nodes is placed between input and output layers, where all three layers are responsible for learning process of the network.

The data were divided randomly into three groups. The first group was considered for training with 23 compounds. The second group was used for validation containing 5 compounds and testing set with 5 compounds. At the end of the training process, it is necessary to evaluate the capability of ANN model in prediction of other data. The validation set is used to monitor the performance of the model during the training phase and to minimize over fitting. Finally the test set is used to evaluate the trained neural network. 

The input vector presented to an ANN is normalized between 0 and 1.

We used the multi-layer perceptron (MLP) network models with back propagation in which weighted sum of inputs and bias term are passed to the activation level through the transfer function to produce the output. Transfer functions can take any form and may be linear or non-linear (20). In this study transfer function in the first layer is the ’S’ shaped logistic sigmoid whose general form is given as   and transfer function in the second layer is linear. In this structure, functions can be well approximated. Back-propagation algorithm based on MATLAB’s Neural Network Toolbox was used for ANN training. In this method, the output response is compared to a desired target response; if the actual response differs from the target response, the network generates an error signal, which is then used to calculate the adjustments that should be made to correct parameter weights, so that the actual output matches the target output. This algorithm is intended to change the weights until the error between output (predicted data) and target (docking data) is minimized.

**Figure 1:**
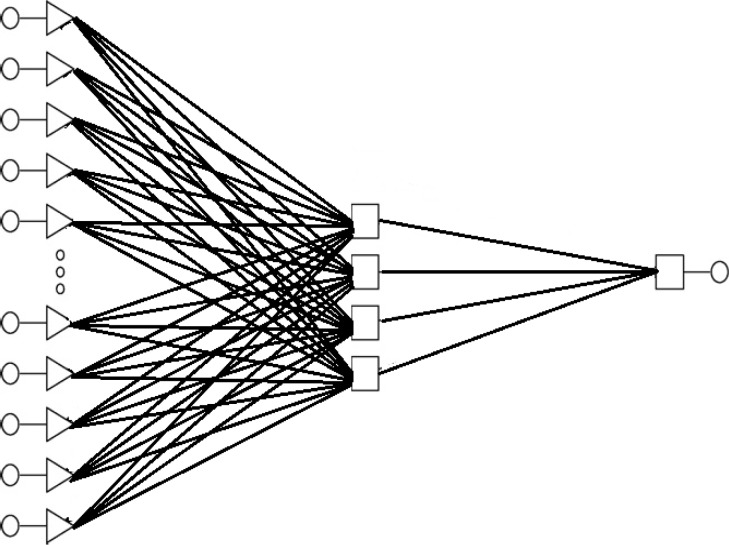
Architecture of artificial neural network predicting binding energy on the basis of selected structural descriptors. Artificial Neural Networks model type: MLP 27-4- 1

**Figure 2:**
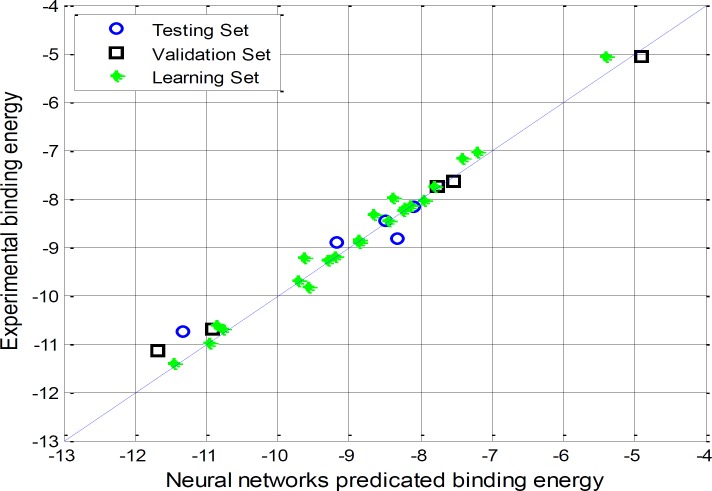
Correlations between the Artificial Neural Networks and docking binding energy

Learning was completed in 150 epochs by back propagation method. In order to decrease the sensitivity predicted results by ANN, to displacement of compounds in different sets; this experiment was done 40 times with diverse selections from training, validation and test data sets. 

Figure 1 represents the architecture of the ANN model used for predictions of binding energy.

## Results

The list of values of the structural parameters of the drugs studied derived from calculation chemistry, reflecting their electronic properties, size (bulkiness), lipophilicity and other 2D and 3D parameters are summarized in Table 4.

Table 3 as Figure 1 shows; we have 27 neurons in the input layer, 4 neurons in hidden layer and 1 neuron in the output layer. Thus the final model possessed a 27-4-1 architecture.

An ANN model was used to correlate binding energy behavior of the set of structurally diverse drugs with their structural descriptors and to create a model useful to prediction of binding energy.

Regression R values measure the correlation between outputs and targets. An R value of 1 means a close relationship while 0 means a random relationship. In Table 1 the correlation coefficients between experimented outputs and predicated outputs are presented. These results are the averages of 40 iterations for each set.

A correlation between docking and ANN binding energy values in learning, validating and testing set is given in Figure 2. 

## Discussion

Results show that Autodock and ANN data have a high correlation. As seen in Table 1, the accuracy of the results increases with augmentation of hidden layer nodes. On the other hand, we achieved a good result and there was no need to increase neurons in hidden layer. Thus model 27-4-1 is a good structure. 

Table 2 shows the information about errors between target and output.

**Table 1:** The standard Pearson-R correlation coefficient between the target and actual output values

Number of neurons in hidden layer	Learning set	Validation set	Testing set
2	0.940	0.902	0.845
3	0.973	0.952	0.930
4	0.973	0.956	0.950

**Table 2:** Statistics of Artificial Neural Networks processing used during the study with 4 neurons in hidden layer

Statistics	Learning set	Validation set	Testing set
Error meana	0.01	0.029	0.053
Error SDb	0.1	0.21	0.3
Abs E Mean_c_	0.095	0.19	0.241
			

Average error of the output variable Standard deviation of errors for the output variable Average absolute error (difference between target and output values) of the output variable

**Table 3:** List of structural parameters employed in ANN analysis

Abbreviation	Description
MW	Molecular weight
Sv	Sum of van der waals volumes C
ISIZ	Information index on molecular size
ZM1	Zagreb m1 index
ZM2	Zagreb m2 index
Qindex	Quadratic index
Pol	Polarity number
TWC	Total walk count
GGI1	Topological charge index
ATS1m	Broto-Moreau autocorecction of a topological structure lag one weighted by atomic mass
ATS1v	Broto-Moreau autocorecction of a topological structure lag one weighted by atomic van der waals
AROM	Aromaticity
AGDD	Averagegeometric distance degree
MAXDN	Maximal electrotopologicalnegative variation
MAXDP	Maximal electrotopological positive variation
MEV	Molecularelectrotopological variation
SPH	Spherosity
ASP	Asphericity
FDI	Folding degree index
Tu	Total size index
ITH	Total information index on leverage content
Ui	Unsaturation index
Hy	Hydrophilic factor
ARR	Aromatic ratio
MR	Molarrefractivity
PSA	Polar surface area
MLOGP	LogP
BE	BindingEnergy (KCal.mol^-1^)

**Table 4:** List of drugs studied, binding energy values and structural parameters

Name	MW	Sv	ISIZ	ZM1	ZM2	Qindex	Pol	TWC	GGI1	ATS1m	ATS1v	AROM	AGDD	MAXDN	MAXDP
Acetaminophen	151.18	12.41	86.439	50	53	6	11	70.3	3	0.64	0.593	0.987	70.162	1.782	3.524
Aspirin	180.17	13.44	92.239	60	66	7	16	86.9	3	0.732	0.61	0.922	79.012	2.781	3.612
Benoxaprofen	301.74	22.85	166.465	112	132	17	32	170	5	0.79	0.683	0.925	179.753	2.532	4.038
Celecoxib	381.41	26.61	212.877	142	166	22	41	215.6	8.5	0.969	0.661	0.954	215.823	5.856	5.125
Diclofenac	296.16	21.08	147.207	94	107	12	27	139.2	4	0.834	0.695	0.984	133.622	2.556	3.827
Diflunisal	250.21	17.75	122.211	92	107	13	28	138.3	4.5	0.811	0.684	0.942	113.926	2.989	5.52
Dup697	411.33	25.58	179.525	124	145	19	35	187.9	6.5	1.19	0.753	0.954	173.698	4.128	5.124
Etodolac	287.39	25.51	226.477	114	140	18	38	181.1	5	0.602	0.602	0.949	195.592	2.487	4.286
Etoricoxib	358.87	27.02	206.131	128	149	19	38	193.1	6.5	0.935	0.697	0.883	198.474	4.145	4.613
Fenoprofen	242.29	20.72	160	88	99	11	24	128.7	3.5	0.653	0.639	0.997	149.165	2.508	3.929
Flurbiprofen	244.28	20.32	153.58	90	104	12	27	134.4	3.5	0.668	0.662	0.962	138.997	2.583	5.772
Ibuprofen	206.31	19.4	166.465	70	77	8	19	100.9	4	0.521	0.584	0.997	151.669	2.438	3.762
Indomethacin	357.81	27.56	219.66	132	158	19	42	204.5	5.5	0.755	0.658	0.91	213.115	2.621	5.987
Ketoprofen	254.3	21.72	166.465	94	108	12	28	139.6	3.5	0.653	0.664	0.898	147.718	2.565	5.244
Ketorolac	211.28	19.09	140.881	86	103	14	22	132.2	2	0.643	0.661	0.901	131.146	1.534	5.246
Lumiracoxib	293.74	22.05	166.465	100	114	13	29	148.4	5	0.755	0.662	0.978	149.781	2.613	5.746
Meclofenamicacid	296.16	21.08	147.207	96	112	13	30	144.5	4	0.834	0.695	0.946	138.029	2.678	4.143
Mefenamicacid	241.31	21.2	166.465	90	104	12	27	134.3	3.5	0.626	0.637	0.944	151.165	2.594	4.124
Meloxicam	351.44	24.19	186.117	126	153	20	41	197.2	6	1.054	0.657	-38.108	176.489	4.814	5.509
Nabumetone	228.31	20.81	166.465	84	95	11	23	123.8	4	0.598	0.627	0.94	169.217	1.431	3.948
Naproxen	230.28	19.72	153.58	86	100	12	26	129.1	4	0.642	0.628	0.94	144.755	2.474	3.916
Nimesulide	310.36	22.22	179.525	106	118	14	28	155.1	6	0.951	0.608	0.63	172.156	4.41	4.348
NS-398	316.42	24.02	219.66	106	118	14	28	155.1	6	0.828	0.564	0.989	204.15	4.359	4.388
Oxaprozin	293.34	24.71	192.75	112	129	15	30	167.9	3.5	0.691	0.661	0.966	197.506	2.527	3.735
Parecoxib	370.46	28.91	240.215	136	160	19	41	207.2	6	0.869	0.653	0.91	249.029	4.785	5.192
Piroxicam	331.38	24.11	186.117	124	150	19	42	193.4	5	0.966	0.653	0.811	188.417	4.823	5.502
Rofecoxib	314.38	24.32	186.117	118	139	18	34	179.8	5	0.911	0.684	0.886	179.386	4.15	5.009
Sulindac	356.44	28.11	226.477	132	156	19	40	202.2	6	0.809	0.684	0.826	231.474	2.611	5.656
Suprofen	260.33	20.21	147.207	90	104	12	25	133.9	3.5	0.786	0.682	0.894	140.782	2.535	5.017
Tolmetin	257.31	21.71	172.974	96	111	13	27	143.8	4.5	0.651	0.627	0.899	172.356	2.574	5.309
Valdecoxib	314.39	24.2	186.117	118	139	18	34	179.8	5	0.925	0.672	0.911	178.467	4.617	4.333
Zileuton	222.34	18.17	140.881	76	87	11	19	113.2	3.5	0.713	0.623	0.685	123.078	2.134	3.728
Zomepirac	243.28	20.12	153.58	92	109	13	28	140.7	3.5	0.676	0.636	0.887	138.427	2.701	5.31

## Conclusion

In present study, a set of 27 descriptors is adopted to build a model to describe docking energy of 33 drugs of diverse chemical structure with antagonistic effects on COX2 enzyme. We built a structure using neural networks which predicts binding energy and developed a multi-layer perceptron artificial neural network (ANN) model, which has been trained by back propagation algorithm. Results show that docking results and ANN data have a high correlation. As presented in Table 1, correlation coefficients for learning, validating and testing sets equaled 0.973, 0.956 and 0.95, respectively. Also the error between the autodock results and ANN data was good. It was shown that ANN is a strong tool for prediction of the binding energy and thus inhibition constants for different drugs in very short period to minimize the amount of time used in virtual screening techniques. 
